# PKCδ and θ Possibly Mediate FSH-Induced Mouse Oocyte Maturation via NOX-ROS-TACE Cascade Signaling Pathway

**DOI:** 10.1371/journal.pone.0111423

**Published:** 2014-10-28

**Authors:** Qian Chen, Wenqiang Zhang, Hao Ran, Lizhao Feng, Hao Yan, Xinyi Mu, Yingying Han, Wei Liu, Guoliang Xia, Chao Wang

**Affiliations:** 1 State Key Laboratory of Agrobiotechnology, College of Biological Sciences, China Agricultural University, Beijing, People’s Republic of China; 2 Department of histology and embryology, Chongqing medical university, Chongqing, People’s Republic of China; 3 College of biological sciences and technology, Beijing Forestry University, Beijing, People’s Republic of China; Inner Mongolia University, China

## Abstract

In mammals, gonadotropins stimulate oocyte maturation via the epidermal growth factor (EGF) network, and the protein kinase C (PKC) signaling pathway mediates this process. Tumor necrosis factor-α converting enzyme (TACE) is an important protein responding to PKC activation. However, the detailed signaling cascade between PKC and TACE in follicle-stimulating hormone (FSH)-induced oocyte maturation *in vitro* remains unclear. In this study, we found that rottlerin (mallotoxin, MTX), the inhibitor of PKC δ and θ, blocked FSH-induced maturation of mouse cumulus-oocyte complexes (COCs) *in vitro*. We further clarified the relationship between two molecules downstream of PKC δ and θ and TACE in COCs: nicotinamide adenine dinucleotide phosphate (NADPH) oxidase (NOX) and its products, reactive oxygen species (ROS). We proved that the respective inhibitors of NOX, ROS and TACE could block FSH-stimulated oocyte maturation dose-dependently, but these inhibitory effects could be reversed partially by amphiregulin (Areg), an EGF family member. Notably, inhibition of PKC δ and θ prevented FSH-induced translocation of two cytosolic components of NOX, p47^phox^ and p67^phox^, to the plasma membrane in cumulus cells. Moreover, FSH-induced TACE activity in cumulus cells was decreased markedly by inhibition of NOX and ROS. In conclusion, PKC δ and θ possibly mediate FSH-induced meiotic resumption in mouse COCs *via* NOX-ROS-TACE signaling pathway.

## Introduction

Oocytes in Graafian follicles of mammalian ovaries arrest at the diplotene stage of meiosis prophase I until a surge of luteinizing hormone (LH) is released before ovulation. Epidermal growth factor (EGF)-like factors, such as amphiregulin (Areg), epiregulin (Ereg) and betacellulin (Btc) secreted by mural granulosa and cumulus cells, are essential for LH-induced oocyte maturation *in vivo* [1]. Reportedly, rapid expression of *Areg* mRNA is induced within 1 h by an ovulatory dose of the LH analog human chorionic gonadotropin (hCG). Areg mimics LH function and induces oocyte maturation and cumulus expansion, and follicle-stimulating hormone (FSH) induces EGF-like growth factors expression in cumulus cells *in vitro*, which in turn induce maturation of cumulus-oocyte complexes (COCs) [2]–[4]. However, the exact mechanism regarding this phenomenon is unclear.

Protein kinase C (PKC) plays important roles in oocyte meiotic resumption, ovulation, egg activation and corpus luteal function [5], [6]. PKC signaling acts in concert with protein kinase A (PKA) to mediate gonadotropin-induced oocyte maturation by activating the EGF receptor (EGFR) signaling pathway in species such as mice, rats and pigs [2], [4], [7]. Previous studies on different cell types showed that several proteins are involved in PKC activated signaling. For example, tumor necrosis factor-α converting enzyme (TACE) [8] is principally responsible for the shedding of Areg, Ereg and Btc [9], [10]. The initiation of EGF-like factors and TACE is mediated directly by gonadotropins in the ovaries [11]–[14]. Generally, the 11 members of PKC family belong to three subtypes based on cofactor requirements: the classical PKC (α, βI, βII and γ), the novel PKC (δ, ε, θ and η) and the atypical PKC (ζ, µ and λ/ι). However, which PKC isoform involved in EGF-like factors and TACE activation remains unknown [15].

Several PKC-related molecules are involved in PKC-mediated TACE activation. The first is reactive oxygen species (ROS), effective second-messenger molecule regulating numerous actions [16], [17]. ROS activates TACE for EGF-like factor shedding, either through modification of a cysteine that coordinates the binding of the inhibitory prodomain [15] or by a post-transcriptional mechanism [18]. The second molecule is nicotinamide adenine dinucleotide phosphate (NADPH) oxidase (NOX), which is the primary enzyme responsible for the production of intracellular ROS, specifically hydrogen peroxide, in many cell types [18], [19]. NOX activation in phagocytes can be induced by a large number of soluble and particulate agents and is dependent on the phosphorylation of the cytosolic proteins p47^phox^ and p67^phox^ [20]. Although the aforementioned proteins are important for mediating PKC signaling in cells, whether all are involved in FSH-induced oocyte maturation *in vitro* and the mechanism by which they react with each other remain unknown.

This study was designed to identify important PKC subfamily members that may be involved in FSH-induced mouse oocyte meiosis *in vitro*. We also clarified the relationships of PKCs with TACE, ROS, and NOX in this process.

## Materials and Methods

### Animals

Immature 21–23-day-old Kunming white female mice (outbreed strain) with a body weight of 12–14 g were used for all experiments. Mice were housed under controlled temperature (23±2°C) and lighting (16 h light and 8 h dark) with food and water provided *ad libitum*. Follicle development was primed by intraperitoneal injection of 5 IU pregnant mare serum gonadotropin (PMSG), and mice were executed by cervical dislocation 44–46 h later.

### Ethics statement

Animal care and use were conducted in accordance with the Institutional Animal Care and Use Committee of China Agricultural University. All experimental procedures were approved by the Institutional Animal Care and Use Committee of China Agricultural University (The Certificate of Beijing Laboratory Animal employee, ID: 18049). All efforts were made to minimize animal suffering.

### Chemicals

All reagents were purchased from Sigma-Aldrich (St. Louis, MO, USA) unless otherwise specified. FSH was prepared as stock solutions in 0.9% (w/v) saline solution, and the final concentration used in culture was 0.05 IU/mL. Areg was prepared as a 10-mg/mL stock solution in distilled phosphate-buffered saline (PBS) containing 0.1% bovine serum albumin (BSA). The specific PKC inhibitor α and β1 Gö6976, the PKC δ and θ inhibitor rottlerin (mallotoxin, MTX) and the NOX inhibitor, diphenyleneiodonium chloride (DPI) were dissolved in dimethylsulfoxide (DMSO) as 10-mM, 10-mM and 1-mM stock solutions, respectively. ROS scavenger 1,3-dimethyl-2-thiourea (DMTU) was dissolved in culture medium at a 30-mM concentration. TAPI-2, the selective TACE inhibitor, was purchased from Enzo Life Sciences, Inc. (Farmingdale, NY, USA) and dissolved in culture medium at a concentration of 20 mM. PI, the pseudosubstrate PKC **ζ** inhibitor, was purchased from Merck (Darmstadt, Germany) and dissolved in culture medium as a 1-mM stock solution. The stock solutions were kept at −20°C and further diluted in culture medium before use.

Both the rabbit anti-p67^phox^ and goat anti-p47^phox^ polyclonal antibodies were purchased from Santa Cruz Biotechnology, Inc. (Santa Cruz, CA, USA).

### Isolation of COCs and *in vitro* culture

Mouse COCs were isolated under a stereomicroscope by puncturing the follicles (300–400 µm in diameter) using a gauge needle in culture medium. COCs of equal size with several layers of cumulus cells were collected. The culture medium was M199 (GIBCO Invitrogen, Carlsbad, CA, USA) containing 4 mM hypoxanthine (HX), 0.23 mM sodium pyruvate, 2 mM glutamine, 3 mg/mL bovine serum albumin, 75 µg/mL penicillin G and 50 µg/mL streptomycin sulfate. The culture medium was equilibrated overnight before usage.

In each experiment, COCs were cultured in 24-well cell culture plates with 1 mL culture medium. COCs were cultured at 37°C in an atmosphere of 5% CO_2_ and 100% humidity. After culture, oocytes were denuded mechanically and assessed for maturation by scoring for germinal vesicles (GV, meiotic arrest), germinal vesicle breakdown (GVB, meiotic resumption) and the first polar body (PB1, the first meiotic maturation). The percentage of GVB (including PB1) per total number of oocytes (% GVB) was calculated.

### Quantitative real-time-PCR (qRT-PCR) analysis

Total RNA of cumulus cells from cultured COCs was isolated and purified from frozen samples using the RNeasy micro-RNA isolation kit (Qiagen, Valencia, CA, USA) according to the manufacturer’s instructions. Reverse transcription was performed directly after RNA isolation using the QuantiTek reverse transcription system (Qiagen). Real-time PCR was then conducted to quantify the steady-state mRNA levels using an ABI 7500 Real-time PCR instrument (Applied Biosystems, Foster City, CA, USA). The results were first normalized to the expression levels of a housekeeping gene, *β-actin*, using the 2^−ΔΔCt^ method [21], and the transcript expression levels were presented as the ratio of the treatment groups to controls. PCR primer sequences were as follows: *Areg*: 5′*-*GGTCTTAGGCTCAGGCCATTA-3′ (forward) and 5′-CGCTTATGGTGGAAACCTCTC-3′ (reverse); *Ereg*: 5′-TTGGGTCTTGACGCTGCTTT-3′ (forward) and 5′-GGATCACGGTTGTGCTGATAA-3′ (reverse); *Btc*: 5′- AATTCTCCACTGTGTGGTAGCA-3′ (forward) and 5′-GGTTTTCACTTTCTGTCTAGGGG-3′ (reverse). To avoid false-positive signals, dissociation curve analyses were performed at the end of the amplification, and the PCR products were subjected to agarose gel electrophoresis to confirm the sizes. The reactions were conducted at least twice.

### TACE activity assay

COCs were cultured for 0, 2 or 4 h under different treatment conditions. Each group contained 300 COCs. For inhibitory studies, COCs were pretreated with inhibitors for 30 min before exposure to FSH. Cumulus cells were collected and washed with PBS. TACE activity in the cell lysates was assayed using the SensoLyte 520 TACE (α-secretase) Activity Assay Kit *Fluorimetric* (ANASPEC).

### Immunofluorescence

COCs cultured for 0 or 1 h under different treatment conditions were collected and washed with PBS. COCs were fixed with 4% (v/v) paraformadehyde, washed with PBS, permeabilized with 2.5% (v/v) Tween-20 and blocked with 10% (v/v) donkey serum. Then, COCs were incubated with a rabbit primary antibody against p67^phox^ or goat primary antibody against p47^phox^ overnight at 4°C. The COCs were washed in PBS and incubated with FITC-labeled donkey anti-rabbit IgG secondary antibody and tetramethyl rhodamine isothiocyanate (TRI/TC)-labeled donkey anti-goat IgG secondary antibody for 1 h. The negative controls for this experiment included COCs exposed to rabbit IgG and goat IgG.

### Statistical analysis

Each oocyte maturation experiment was conducted at least three times with a minimum of 60 oocytes per group. Results were expressed as means ± standard error of the mean (SEM). All proportional data were subjected to arcsine transformation and analyzed using the least squares method with SAS software. A *p-*value<0.05 was considered to indicate statistical significance.

## Results

### 1. Areg is involved in PKC δ- and θ-mediated FSH-induced meiotic resumption in COCs

**Figure 1:**
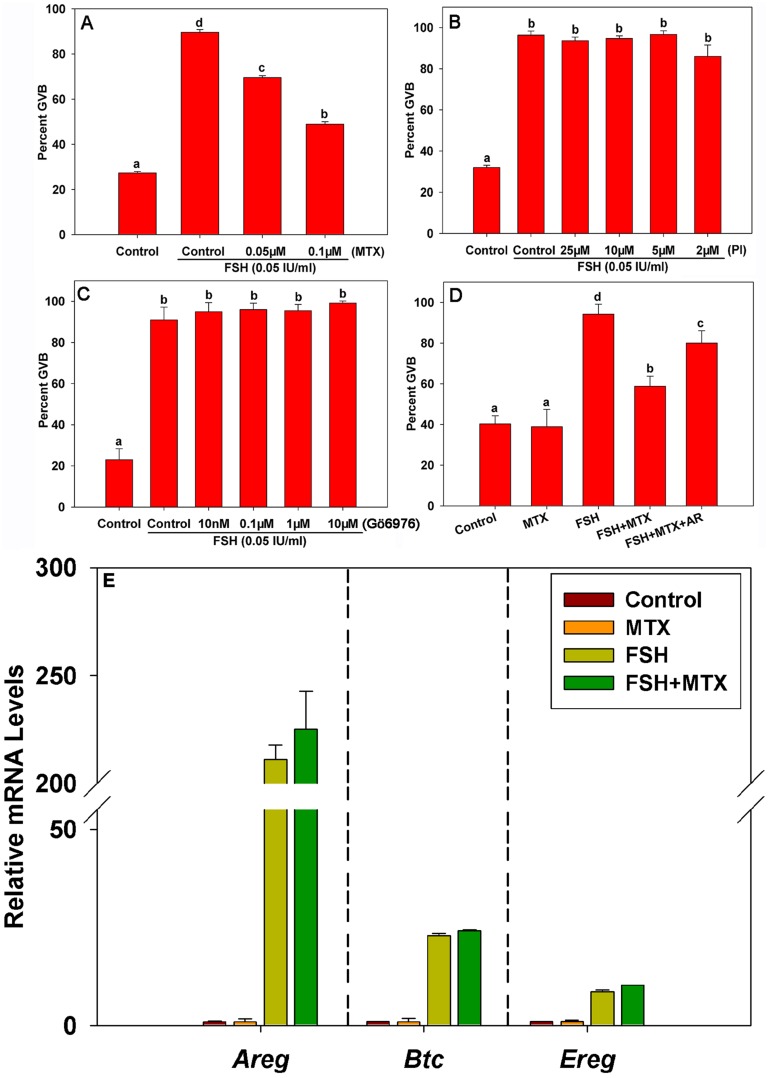
The effect of Areg on PKC δ- and θ-mediated FSH-induced meiotic resumption in COCs. The germinal vesicle breakdown (GVB) ratio of COCs cultured in FSH-supplemented media with or without the PKC δ and θ inhibitor rottlerin (MTX) (**A**), PKC ζ pseudosubstrate inhibitor (PI) (**B**), or PKC α and βI inhibitor Gö6976 (**C**) for 22–24 h. The GVB ratio of COCs cultured in media supplemented with either 0.1 µM MTX, 0.05 IU/mL FSH, MTX plus FSH, or MTX plus FSH plus 100 ng/mL Areg for 22–24 h (**D**). After COCs were cultured in media with either FSH (0.05 IU/mL) and/or 0.1 µM MTX for 4 h, the mRNA levels of *Areg*, *Ereg* and *Btc* in cumulus cells were detected using real-time PCR (**E**). Letters (a–d) on different columns in each chart indicate significant difference between groups (*p*<0.05). AR: Areg.

In this study, FSH strongly induced meiotic resumption in mouse oocytes after 22–24 h of *in vitro* culture compared with the control group (Fig. 1A–1D). However, the effect of FSH on oocyte maturation was inhibited dose-dependently by the specific PKC δ and θ inhibitor MTX (Fig. 1A). Conversely, neither the specific PKC α and β1 inhibitor Gö6976 nor the PKC **ζ** pseudosubstrate inhibitor (Pi) prevented FSH-induced meiotic resumption (Fig. 1B, 1C). Additionally, administration of the EGF-like peptide Areg (100 ng/mL) reversed the suppressive effect of MTX (Fig. 1D) in our culture system, indicating that Areg is involved in PKC δ and θ-mediated FSH-induced mouse COC maturation. To investigate the mechanism of PKC δ and θ regulation of EGF-like factors function, we measured the expression levels of transcripts encoding the EGF-like factors Areg, Ereg and Btc in COCs. The results showed that FSH significantly promoted the expression levels of EGF-like factor mRNAs after 4 h of culture (Fig. 1E), whereas MTX exerted no effect on the expression levels of these genes. Therefore, PKC δ- and θ-mediated FSH-induced oocyte maturation was not based on transcriptional regulation of EGF-like growth factors.

### 2. NOX and its ROS products participate in PKC δ- and θ-mediated FSH-induced meiotic resumption in COCs

**Figure 2:**
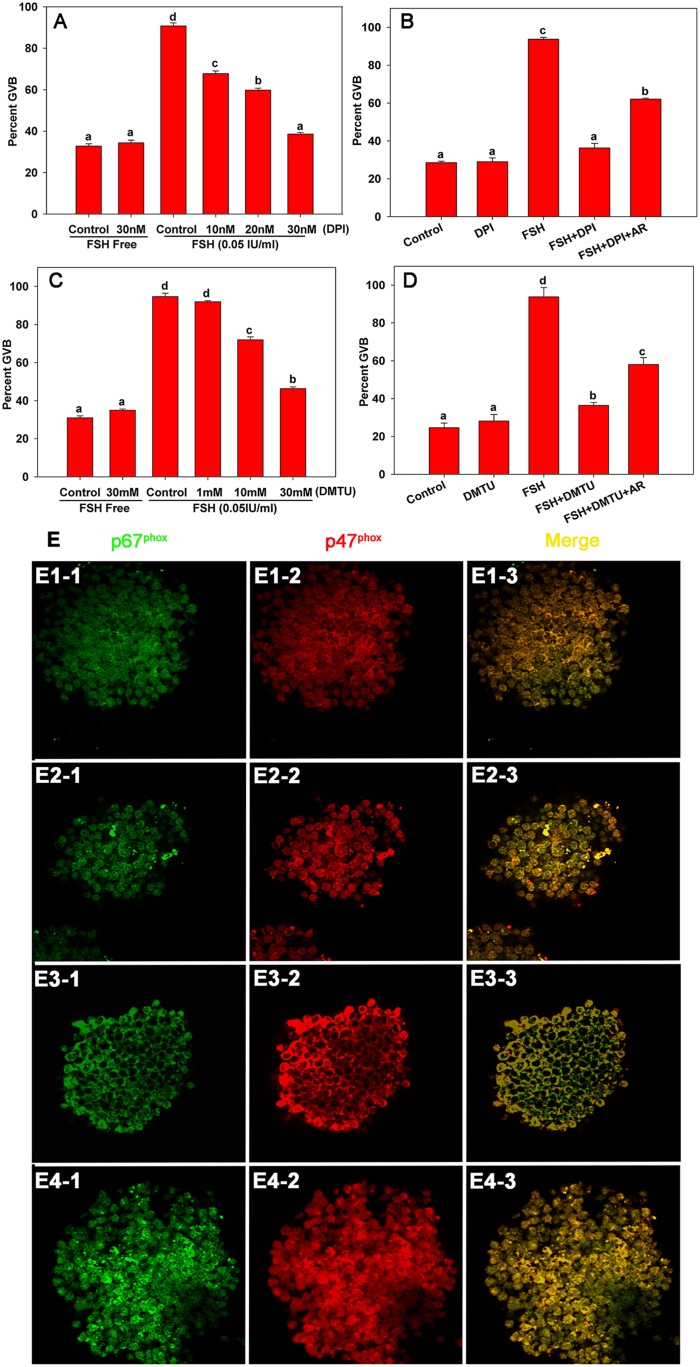
The effect of NOX and its ROS products on PKC δ- and θ-mediated FSH-induced meiotic resumption in COCs. COCs were cultured respectively in the following media: (1) FSH-supplemented media with or without NOX inhibitor diphenyleneiodonium chloride (DPI), ROS scavenger 1,3-dimethyl-2-thiourea (DMTU) for 22–24 h (**A and C**); (2) media supplemented with either 30 nM DPI, 0.05 IU/mL FSH, DPI plus FSH, or DPI plus FSH plus 100 ng/mL Areg (AR) for 22–24 h (**B**); (3) media supplemented with either 30 mM DMTU, 0.05 IU/mL FSH, DMTU plus FSH, or DMTU plus FSH plus 100 ng/mL Areg for 22–24 h (**D**). Figure E showed the expression pattern of p67^phox^ and p47^phox^ in COCs cultured under different treatment conditions at the indicated time points. Samples were stained with anti-p67^phox^ antibodies (E1-1–E4-1) or anti-p47^phox^ antibodies (E1-2–E4-2), and the reactions were developed to produce either a red (p47^phox^) or green (p67^phox^) color. E1-1–E1-3: COCs cultured in HX medium for 0 h. E2-1–E2-3: COCs cultured in HX medium for 1 h. E3-1–E3-3: COCs cultured with FSH (0.05 IU/mL) in HX medium for 1 h. E4-1–E4-3: COCs cultured with FSH (0.05 IU/mL) and 0.1 µM rottlerin (MTX) in HX medium for 1 h. Letters (a–d) on different columns in each chart indicate significant difference between groups (*p*<0.05). AR: Areg.

Existing studies indicated that NOX and its ROS products participate in post-transcriptional regulation of EGF-like growth factors [18], [19]. Accordingly, we speculated that PKC δ and θ might mediate FSH-induced oocyte maturation by post-transcriptional activation of EGF-like growth factors. Therefore, we first explored whether NOX and ROS were involved in FSH-induced meiotic resumption in COCs. As shown in Figure 2A, after supplementing the COC culture media with the NOX inhibitor DPI, FSH-induced oocyte maturation was blocked significantly in a dose-dependent manner. After treating COCs with the ROS scavenger DMTU, FSH-induced mouse oocyte GVB was blocked dose-dependently (Fig. 2C). However, the inhibiting effect of DPI and DMTU could be recovered partially by the addition of Areg (Fig. 2B, 2D). These results indicated that NOX and ROS are involved in FSH-induced mouse COC maturation *in vitro*. Since phagocytic NOX generates ROS when the cytosolic components (such as p47^phox^ and p67^phox^) translocate to the plasma membrane to form the complete enzyme NOX together with gp91^phox^ [22], [23], we further examined whether FSH caused NOX activation in COCs via a similar mechanism to that in phagocytes. As show by immunofluorescence analysis (Fig. 2 E), FSH induced p47^phox^ and p67^phox^ translocation to the plasma membrane in cumulus cells when COCs were cultured for 1 h (Fig. 2 E3-1, E3-2) compared with the control (Fig. 2 E1-1, E1-2). However, the translocation of p47^phox^ and p67^phox^ was prevented by the addition of MTX in cumulus cells (Fig. 2 E4-1, E4-2).

### 3. NOX and its ROS products mediate TACE activity during FSH-induced meiotic resumption in mouse COCs

**Figure 3:**
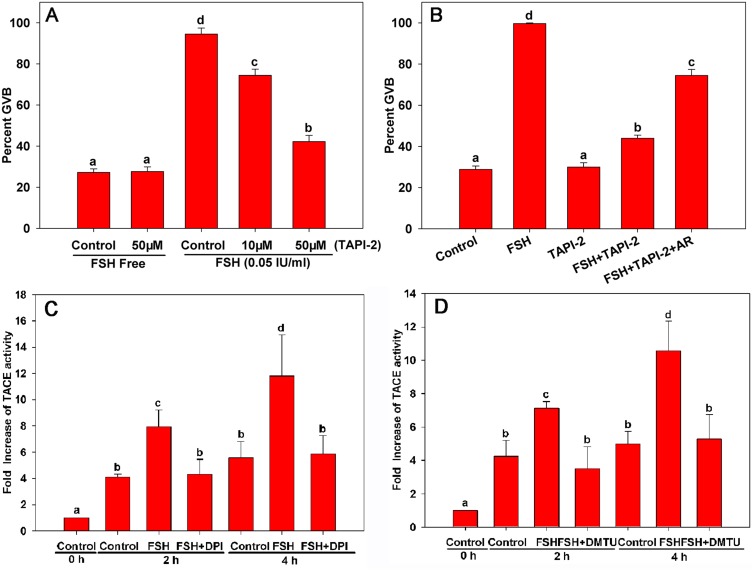
The effect of NOX and its ROS products on TACE activity during FSH-induced meiotic resumption in mouse COCs. COCs were cultured respectively in the following media: (**A**) FSH-supplemented media with or without TACE selective inhibitor TAPI-2 for 22–24 h; (**B**) media supplemented with 50 µM TAPI-2, 0.05 IU/mL FSH, TAPI-2 plus FSH, or TAPI-2 plus FSH plus 100 ng/mL Areg for 22–24 h. Figures **C** and **D** indicate the TACE activity in cumulus cells of COCs cultured in FSH-supplemented media with or without NOX inhibitor diphenyleneiodonium chloride (DPI, 30 nM), ROS scavenger 1,3-dimethyl-2-thiourea (DMTU, 30 mM) for 0, 2 or 4 h. Letters (a–d) on different columns in each chart indicate a significant difference between groups (*p*<0.05). AR: Areg.

Reportedly, TACE is mediated directly by gonadotropins in ovaries and is important for the shedding of pro-EGF-like factors to form active EGFR ligands [11], [12]. Therefore, we investigated whether TACE is involved in FSH-induced meiotic resumption in mouse COCs. As shown in Figure 3A, the TACE inhibitor TAPI-2 alone had no effect on meiotic resumption in COCs. However, FSH-induced meiotic resumption in COCs was inhibited dose-dependently by TAPI-2, and Areg reversed the suppressive effect of TAPI-2 (Fig. 3B). After analyzing the temporal changes in TACE activity in cumulus cells of *in vitro*-cultured COCs, TACE activity was increased significantly after COCs were cultured with FSH for 2 h, as compared with the control group (Fig. 3C, 3D).

Additionally, to prove that NOX could modulate TACE activity via ROS in cumulus cells during FSH-induced oocyte maturation, we examined the levels of TACE protein activity in cumulus cells of COCs cultured with FSH in the presence of the NOX inhibitor DPI and the ROS scavenger DMTU, respectively. As shown in Figures 3C and 3D, TACE activity levels in cumulus cells decreased significantly when COCs were cultured with these antagonists for 2 or 4 h, which confirmed that NOX and its ROS products mediated TACE activity during FSH-induced meiotic resumption in mouse COCs.

## Discussion

PKC is essential for gonadotropin-induced oocyte maturation [2], [4], [13], [24]. For example, PKC α and β1 participate in FSH-induced follicle-enclosed oocyte (FEO) maturation [7]. In this study, based on all PKC isoforms examined, we confirmed that the novel PKC isoforms PKC δ and θ, but not the classical PKC isoforms PKC α and β1 or the atypical PKC isoform PKC ζ, were involved in FSH-induced COC maturation. Our results are consistent with reports that PKC δ is involved in mouse and *Xenopus laevis* oocyte meiotic maturation and egg activation [25], [26]. We were unable to explore the role of the remaining six PKC isoforms in FSH-induced COC maturation since specific inhibitors are currently unavailable. Functional variances in PKC α and β1 were observed between FEOs and COCs during meiotic resumption, which may be due to the different culture models [7]. However, the exact mechanism needs further investigation. In addition, because MTX concentration may affect its action and have other actions on cells *in vitro*, independently of direct PKC inhibition [27], excluding the possibility of an indirect MTX effect on PKC δ and θ is difficult at the concentration applied in our study. Therefore, although we are confident in the results derived from our experiments, more specific inhibitor molecules are needed for precise conclusion regarding the specific PKC subtypes involved in FSH-induced oocyte meiosis.

The EGF network is essential for gonadotropin-induced oocyte maturation, cumulus expansion and ovulation [28], [29]. The PKC signaling pathway participates in EGF network activation, thus resulting in meiotic resumption [4], [13]. Our study indicated that EGF-like factors might participate in novel PKC signaling processes during FSH-induced mouse COC maturation. However, the PKC δ and θ inhibitor had no effect on EGF-like factor mRNA expression, which is consistent with previous studies [4], [13], [30]. Therefore, we hypothesized that PKC may participate in EGFR activation via a transcription-independent mechanism. The EGF network signaling mediated by PKC δ and θ might be activated by the release of mature receptor ligands from their membrane-anchored precursor forms, a process referred to as ectodomain shedding [13], [15], [30].

Previous studies indicated that ROS plays an important role in oocyte maturation [31], [32]. Low levels of ROS may act as a “trigger” of oocyte maturation following the LH surge, whereas antioxidants can inhibit meiotic resumption in COCs and denuded oocytes [3]. In addition, many NOX family members are the predominant contributors of ROS in many cellular systems [19], [22]. Importantly, LH can induce NOX-dependent ROS production in the ovary of immature pseudopregnant mice [33]. The NOX complex was originally identified and characterized in phagocytes. The classical neutrophil NOX comprises a catalytic subunit gp91^phox^, which in conjunction with the p22^phox^ subunit forms a membrane-bound heterodimer. Additionally, a number of cytosolic regulatory subunits, such as p67^phox^ and p47^phox^, are required for enzyme activation [23], [34]. Recently, Nox2 and Nox4, the homologues of subunit gp91^phox^, were shown to generate ROS in the ovary. Upon NOX activation, these cytosolic subunits translocate to the membrane and assemble with the membrane components in a highly regulated process [8]. Accordingly, we found that NOX and ROS participate in FSH-induced COC maturation *in vitro*, suggesting that both NOX and its ROS products are essential to FSH-induced COC maturation.

In phagocytic and epithelial cells, the PKC-phosphorylated cofactor p47^phox^ causes translocation of the p67^phox^/p47^phox^ complex from the cytoplasm to the plasma membrane, where the complex associates with the Nox2 membrane component to achieve maturation and activation of NOX [35]. We showed FSH activated NOX by a similar mechanism in cumulus cell phagocytes, and FSH-induced translocation of the p67^phox^/p47^phox^ complex was mediated via novel PKC signaling. Hence, these results implicated a novel role for PKC in FSH-induced meiotic resumption in mouse COCs mediated by NOX and ROS.

Previous studies have identified TACE as the major EGFR ligand sheddase of Areg and Ereg [9]. TACE is expressed in mouse, rat and porcine cumulus cells during the ovulatory process. In addition, the protein expression level and activity of TACE are increased significantly during gonadotropin-induced porcine oocyte maturation [13], [30]. In the present study, oocyte maturation was inhibited by the TACE selective inhibitor TAPI-2 and reversed by Areg, suggesting that activation of TACE is related to EGF-like factors during FSH-induced oocyte maturation. Generally, TACE is synthesized in a latent form containing an inhibitory N-terminal prodomain masking the catalytic domain; ROS directly activates TACE by removing the inhibitory prodomain in monocytic cells and T cells [18], [36]. Therefore, we further studied the exact relationship between ROS and TACE in this process. TACE activity levels increased rapidly after treatment with FSH for 2 h. The NOX inhibitor DPI and ROS scavenger DMTU inhibited this increase in TACE activity, resulting in suppression of FSH-stimulated oocyte maturation in this study. These results demonstrated that NOX and its ROS products mediate TACE activity in FSH-induced oocyte maturation.

**Figure 4:**
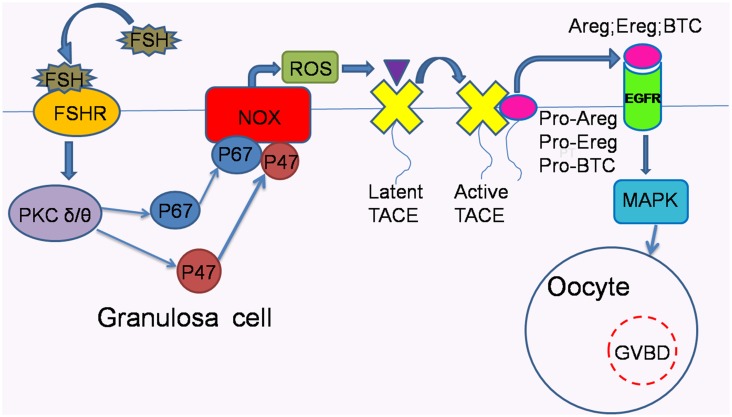
Possible signaling pathways that mediate FSH-induced meiotic resumption in COCs *in vitro*. FSH acts through the downstream mediators PKC δ and θ, which recruit the cytosolic components p47^phox^ and p67^phox^ to the plasma membrane, allowing their combination with membrane components of NOX to form an active enzyme system to generate ROS. Then, ROS activate TACE by oxidizing the inhibitory prodomain to expose the catalytic domain. Next, the activated TACE via ROS cleaves pro-EGF-like factors into soluble EGFR ligands, which bind to and activate EGFR in an autocrine mode, thereby resulting in oocyte maturation.

In conclusion, to better understand the reciprocal relationship among proteins investigated in our study compared with previous studies, a hypothetical schematic is provided in Figure 4, indicating that PKC δ and θ possibly recruit p47^phox^ and p67^phox^, the cytosolic components of NOX, to the plasma membrane to assemble with membrane components. Then, the assembled components activate NOX to release ROS and consequently activate TACE. As a result of the activated shedding function of TACE, pro-EGF-like factors are cleaved into soluble EGFR ligands, which bind to and activate EGFR, leading to oocyte maturation.
